# Deep learning-based defacing tool for CT angiography: CTA-DEFACE

**DOI:** 10.1186/s41747-024-00510-9

**Published:** 2024-10-09

**Authors:** Mustafa Ahmed Mahmutoglu, Aditya Rastogi, Marianne Schell, Martha Foltyn-Dumitru, Michael Baumgartner, Klaus Hermann Maier-Hein, Katerina Deike-Hofmann, Alexander Radbruch, Martin Bendszus, Gianluca Brugnara, Philipp Vollmuth

**Affiliations:** 1 Department of Neuroradiology, Heidelberg University Hospital, Heidelberg, Germany; 2 Division for Computational Neuroimaging, Heidelberg University Hospital, Heidelberg, Germany; 3 Division for Medical Image Computing, German Cancer Research Center, Heidelberg, Germany; 4 Helmholtz Imaging, Heidelberg, Germany; 5 Faculty of Mathematics and Computer Science, Heidelberg University, Heidelberg, Germany; 6 Department of Neuroradiology, Bonn University Hospital, Bonn, Germany; 7 Clinical Neuroimaging Group, German Center for Neurodegenerative Diseases, DZNE, Bonn, Germany

**Keywords:** Artificial intelligence, Computed tomography angiography, Data anonymization, Image processing (computer-assisted), Neural network (computer)

## Abstract

**Abstract:**

The growing use of artificial neural network (ANN) tools for computed tomography angiography (CTA) data analysis underscores the necessity for elevated data protection measures. We aimed to establish an automated defacing pipeline for CTA data. In this retrospective study, CTA data from multi-institutional cohorts were utilized to annotate facemasks (*n* = 100) and train an ANN model, subsequently tested on an external institution’s dataset (*n* = 50) and compared to a publicly available defacing algorithm. Face detection (MTCNN) and verification (FaceNet) networks were applied to measure the similarity between the original and defaced CTA images. Dice similarity coefficient (DSC), face detection probability, and face similarity measures were calculated to evaluate model performance. The CTA-DEFACE model effectively segmented soft face tissue in CTA data achieving a DSC of 0.94 ± 0.02 (mean ± standard deviation) on the test set. Our model was benchmarked against a publicly available defacing algorithm. After applying face detection and verification networks, our model showed substantially reduced face detection probability (*p* < 0.001) and similarity to the original CTA image (*p* < 0.001). The CTA-DEFACE model enabled robust and precise defacing of CTA data. The trained network is publicly accessible at www.github.com/neuroAI-HD/CTA-DEFACE.

**Relevance statement:**

The ANN model CTA-DEFACE, developed for automatic defacing of CT angiography images, achieves significantly lower face detection probabilities and greater dissimilarity from the original images compared to a publicly available model. The algorithm has been externally validated and is publicly accessible.

**Key Points:**

- The developed ANN model (CTA-DEFACE) automatically generates facemasks for CT angiography images.
- CTA-DEFACE offers superior deidentification capabilities compared to a publicly available model.
- By means of graphics processing unit optimization, our model ensures rapid processing of medical images.
- Our model underwent external validation, underscoring its reliability for real-world application.

**Graphical Abstract:**

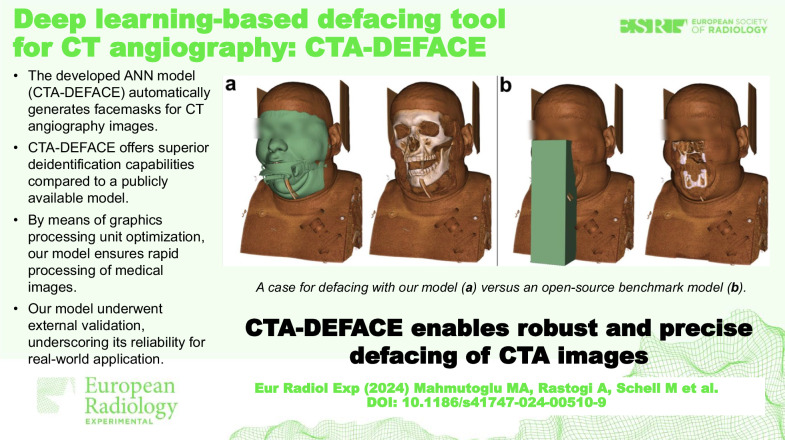

## Background

Computed tomography angiography (CTA) plays a crucial role in evaluating patients with vascular malformations, aneurysms, and tumors, and notably in diagnosing vessel occlusion in acute ischemic stroke. Machine/deep learning technology is increasingly applied to CTA to assess cervical artery anatomy [1] and stenosis [2], radiomics signatures of carotid plaques [3] and to develop automated tools for detecting vessel occlusions [4–7]. This last task often relies on large datasets acquired through data-sharing initiatives. Additionally, the clinical use of such tools may necessitate the sharing of patient images, emphasizing the need for robust data protection protocols.

A critical aspect of deidentifying medical images is the removal or distortion of identifiable facial features [8–10]. While numerous defacing tools are available for magnetic resonance imaging [11–13], they are less available for computed tomography (CT) or positron emission tomography [14–16].

We developed a neural network-based approach for defacing CTA images (CTA-DEFACE), based on the nnU-Net framework [17] and compared our CTA-DEFACE model against the publicly available facemask generator function from the ICHSEG library [18].

## Methods

### Dataset

This retrospective multicenter study was approved by the local ethics committee. CTA data from two cohorts for model training and a third distinct cohort for testing was used. Fifty patients previously treated at Heidelberg University Hospital (cohort 1) and 50 patients from three primary/secondary care hospitals of the regional stroke consortium Rhein-Neckar with acute teleneurology/teleradiology coverage through the Heidelberg University Hospital (cohort 2), were used for model training. These patients were diagnosed with acute ischemic stroke and CTA-confirmed vessel occlusion. Testing involved 50 patients who underwent CTA for suspected acute ischemic stroke at Bonn University Hospital’s Department of Neuroradiology (cohort 3).

Cohorts 1 and 2 were partitioned equally for a balanced representation of each cohort in both training processes. Scanner and acquisition parameters are depicted in Supplementary Table 1, and patient demographics are in Supplementary Tables 2 and 3.

### Study design

**Fig. 1:**
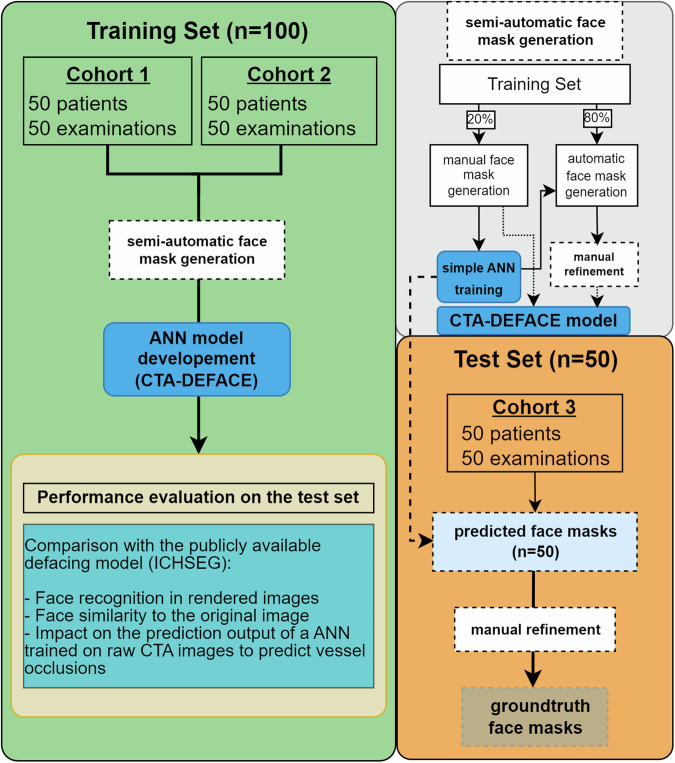
Flowchart of the procedures for CTA-DEFACE and model training and external testing

Figure 1 shows the flowchart of model training and testing. The state-of-the-art nnU-Net was used for training, automatically configuring hyperparameters based on the dataset characteristics [17]. The model was trained on NVIDIA DGX A100 (NVIDIA, Santa Clara, CA, USA) using 2 A100-SXM4 graphics processing units (GPUs) of 40GB each with AMD EPYC 7742 64-Core Processor and 1 TB RAM. The inference runtimes for predictions were measured on a local workstation with an Intel Xeon E5/Core i7 3.1 GHz CPU and NVIDIA TITAN RTX GPU.

### Model development

To train the CTA-DEFACE model, the entire training dataset (*n* = 100) was divided into two equal and disjoint sets from both HD and FAST cohorts (*n* = 50 each). Face mask generation for CTA data was done in Slicer 3D (version 5.4.0), annotating soft face tissue from forehead to chin, including the nose, lips, and masseter muscles. An initial nnU-Net was trained on 20% of the training set (ten patients each from cohorts 1 and 2). Subsequently, this model predicted facemasks from the remaining 80% of the training set and the complete test set. These predictions were manually refined to generate ground truth facemasks. The final nnU-Net training was done using the entire CTA dataset (*n* = 100). The resulting predicted facemasks on the external test set (cohort 3), were compared with the ground truth.

### Face detection, recognition, and validation

To validate our model, we utilized the publicly available CT defacing tool “ct_face_mask” from the “ICHSEG” package in R [18] for comparative analysis. Predicted facemasks from ICHSEG and our model on the test set were replaced with the 10th percentile value of the original image to represent void space. The processing time for face segmentation was measured for both methods.

CTA images from the test set were visualized in Slicer 3D (version 5.4.0) with “CT-Muscle” preset. Rendered images were captured from an anterior viewpoint for the original image and the defaced versions with ICHSEG and CTA-DEFACE. Examples of each algorithm are illustrated in Supplementary Fig. 1.

Unlike classical segmentation tasks, defacing algorithms cannot be compared with traditional metrics like dice similarity coefficient (DSC) due to the lack of actual ground truth. Therefore, to compare ICHSEG and CTA-DEFACE, we employed two neural networks from previous studies [16, 19]. Face detection in rendered images was conducted using a multitask cascaded convolutional neural network (MTCNN) [20], which integrates three convolutional neural network structures for face recognition, bounding box regression, and facial landmark localization. Further validation involved quantifying the face identifiability of CTA images before and after defacing. If a face was detected by MTCNN, FaceNet [21] was used to extract the face embedding vector to verify whether the face matched the rendered original image. Face embedding vectors were calculated by FaceNet on the rendered original CTA image and after defacing with ICHSEG and CTA-DEFACE models. We calculated the Euclidean distance, a measure of similarity, between the embeddings of the original CTA image and the images produced by two defacing strategies (less distance representing a greater similarity).

### Statistical analysis

Statistical analyses were performed using Python (version 3.8.13) and R (version 4.0.5). The performance of each model was assessed on the cohort 3 test set. Face detection probabilities, calculated on the captions of rendered images, underwent analysis using a nonparametric Friedman rank sum test with a post-hoc pairwise Wilcoxon signed-rang exact test with Bonferroni correction. Euclidian distance for face embedding vectors was computed to examine similarities. Paired *t*-tests compared the defacing algorithms. If normal distributions were met, descriptive statistics were provided in terms of mean and standard deviation.

## Results

### CTA-DEFACE model characteristics

**Fig. 2:**
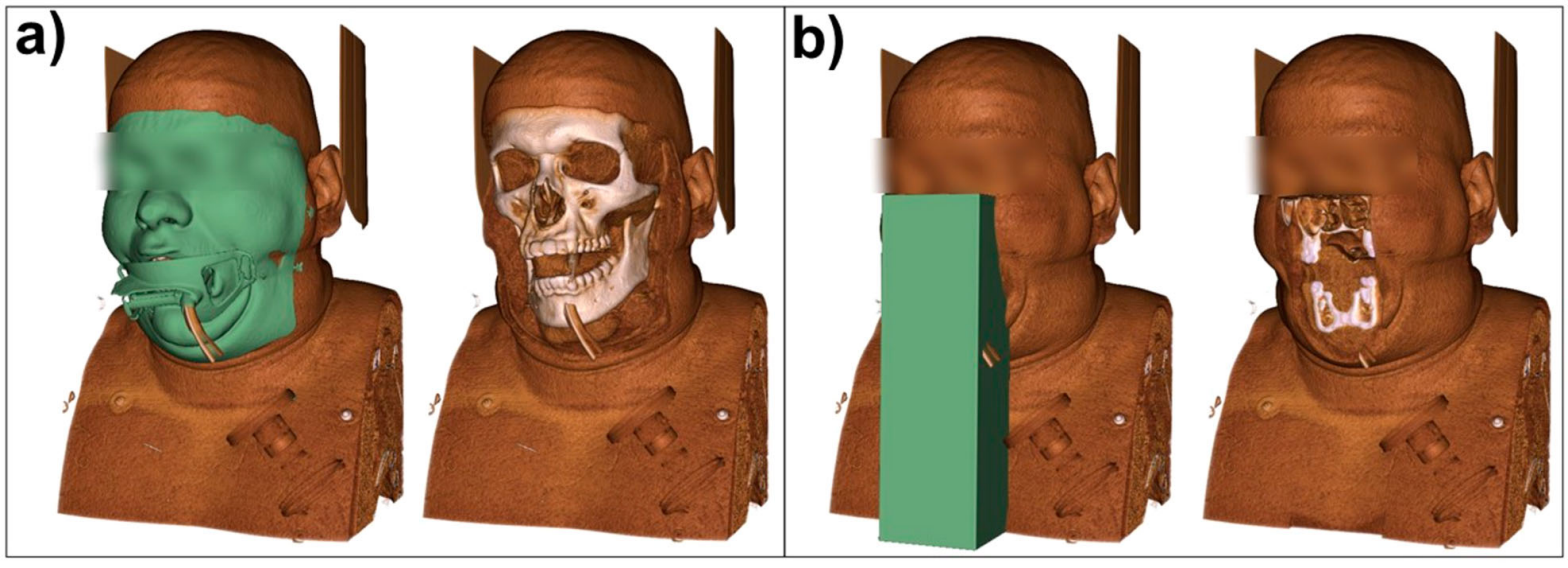
An illustrative case from the test set for CTA-DEFACE (**a**) and the “ct_face_mask” function from the ICHSEG library (**b**). Illustrations are rendered images of CT volumes in Slicer 3D software with the “CT-Muscle” preset. Left: predicted segmentation masks. Right: volume rendering after subtracting the facemask and replacing it with the 10th percentile of the HU value of the image to represent empty air. Eyes are blurred for anonymization purposes

The facemask generated by CTA-DEFACE covers soft tissue and skin from the forehead (above the frontal sinus) to the chin (at the level of hyoid bone), which includes soft tissue around the eyes, nose, masseter muscles, and lips. In cases where external devices are present, such as protective glass or an intubation tube, they are also depicted by the segmentation mask. In contrast, the ICHSEG library’s “ct_face_mask” function predicts a rectangular prism covering the mouth and nose (Fig. 2).

### Segmentation and face recognition metrics

The CTA-DEFACE model achieved a DSC of 0.94 ± 0.02 (mean ± standard deviation) on ground truth facemasks. For each test case, the average face segmentation time was 0.2 ± 0.1 min (mean ± standard deviation) with the CTA-DEFACE model and 36.3 ± 9.2 min with ICHSEG.

**Fig. 3:**
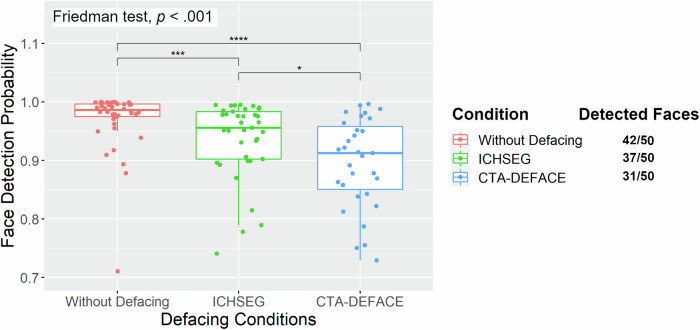
Face detection probabilities by multitask cascaded convolutional neural network (MTCNN) on the rendered CTA images are illustrated in boxplots for the original CTA volume image (left), defaced image with ICHSEG (center) and defaced image with CTA-DEFACE (right). Undetected faces were excluded. The number of detected faces is indicated next to each method (on the right). Nonparametric Friedman rank sum test with post-hoc pairwise comparison using Wilcoxon signed rand exact were calculated. CTA, Computed tomography angiography, *p = 0.04, ***p < 0.001, ****p < 0.0001

Faces were detected by the MTCNN network on 42/50 (84%, 95% confidence interval: 71–93%) of original images without defacing, 37/50 (74%, 95% confidence interval: 60–85%) of images defaced with ICHSEG, and 31/50 (62%, 95% confidence interval: 47–75%) of images defaced with CTA-DEFACE (Fig. 3).

**Fig. 4:**
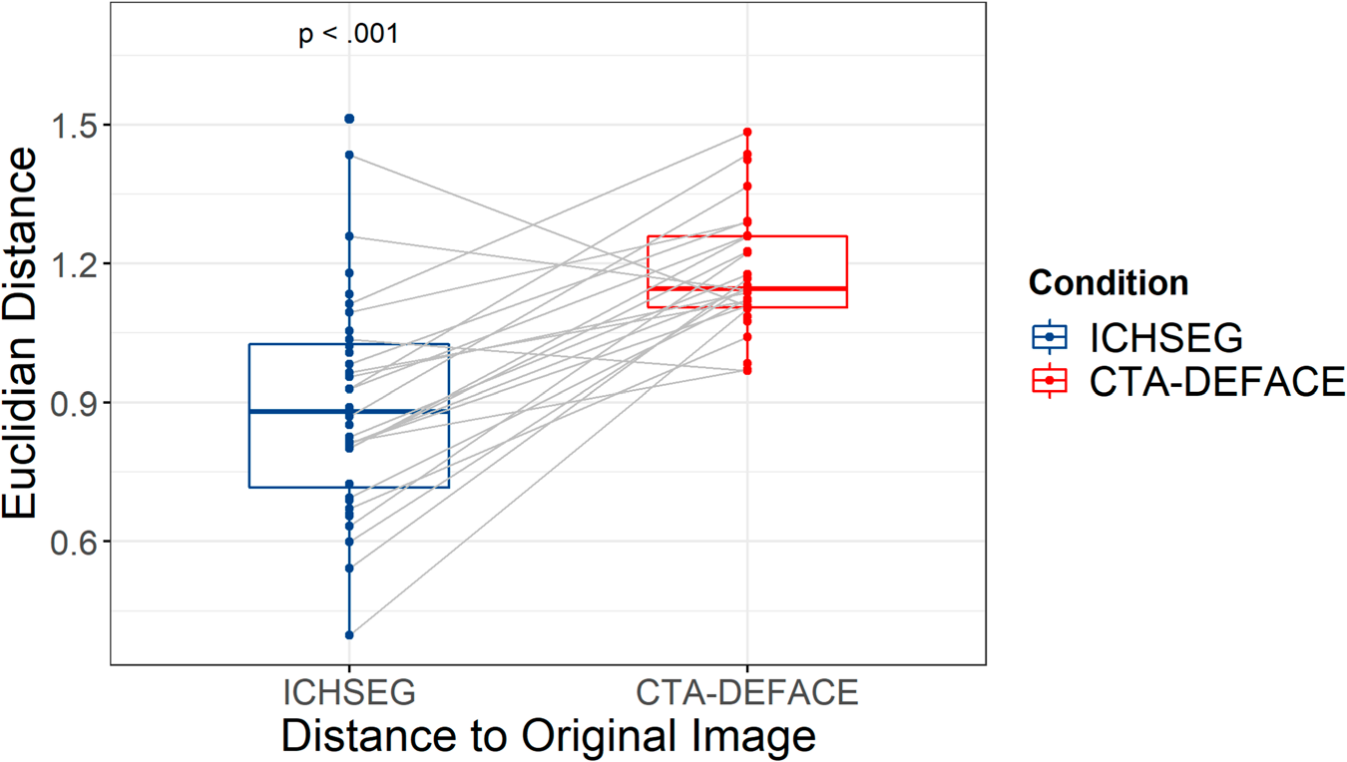
Euclidian distance of face embedding vectors between the original CTA images and defaced images (ICHSEG *versus* CTA-DEFACE) are shown as boxplots. Lower values indicate greater similarity to the original image. The paired *t*-test *p*-value between groups is displayed in the top left. CTA, Computed tomography angiography

Face embedding vectors after defacing with our model showed a significantly increased distance from the original image (Fig. 4).

## Discussion

Reidentification of individuals in brain imaging is highly accurate using face-recognition software [8–10], posing challenges for head CT data in both research and clinical applications. We developed a defacing model (CTA-DEFACE) for CTA data, based on the state-of-the-art segmentation algorithm nnU-Net [17], which automatically generates an anatomical facemask from the forehead to the chin covering the facial soft tissue. We compared our model to a publicly available defacing function “ct_face_mask” from the ICHSEG library [18], which uses a rectangular prism to cover the mouth and nose. The CTA-DEFACE model resulted in significantly lower face detection probabilities and higher dissimilarity to the original image compared to ICHSEG. Furthermore, our model deidentified patient faces faster by leveraging GPU parallel computation.

The performance of defacing algorithms is difficult to compare with traditional metrics such as DSC since an actual ground truth does not exist. Recent algorithms for CT data have already adopted an “anatomical” approach by segmenting facial soft tissue [14], but comparing the correctness of these segmentations of those algorithms is not meaningful. We chose ICHSEG for comparison due to its different defacing strategy [18]. When segmentation masks were replaced by void space, our model revealed significantly lower face detection probabilities and higher dissimilarity to the original image, suggesting that removing more anatomical facial features than only mouth and nose is necessary for robust de-identification.

The application of machine learning models in the medical field, particularly for the head and neck, remains limited. Examples include automated detection of brain hemorrhage [22, 23] and intracranial thrombus in vessels [22–25]. Similar limitations affect defacing tools, as the inclusion of facial features depends on various technical factors such as: (i) scanned area, (ii) slice thickness, (iii) presence of foreign materials like eye protection, (iv) motion artifacts, (v) beam-hardening, and (vi) patient positioning. These factors need to be addressed when applying automated defacing tools.

With respect to data protection regulations, the integration of automated defacing tools into workflows or preprocessing pipelines of commercially available stroke detection programs should consider the fact that defacing medical images removes a substantial portion of facial tissue, altering image characteristics (Supplementary Fig. 2). Therefore, to integrate the CTA-DEFACE model or other defacing protocols into automated stroke detection pipelines, it may be necessary to either retrain the stroke detection models on defaced volumes or perform defacing in the postprocessing.

This study has limitations. First, the number of patients is limited, albeit from different cohorts. The effective de-identification capability of CTA-DEFACE was demonstrated on an external dataset obtained from a different CT vendor. However, a larger dataset is necessary to further evaluate the reproducibility of our results. Second, only CTA data was addressed. Future studies should validate our model including unenhanced and other contrast-enhanced head/neck CT protocols.

In conclusion, our results show that the CTA-DEFACE model effectively segments and removes facial soft tissue from CTA images faster than a publicly available CT defacing method, resulting in significantly lower face detection probabilities and higher dissimilarity to the original image. Future research should evaluate the potential of training algorithms (*e.g*., stroke algorithms) on defaced data.

## Supplementary information

**Additional file 1: Supplementary Table 1:** Computed tomography angiography imaging features are depicted. **Supplementary Table 2:** Patient demographics in Cohort 1, 2 and 3 for CTA-DEFACE model training and testing. Pearson’s chi-squared test was used for comparing categorical variables and Kruskal Wallis test was used for comparing continuous variables between the training and test set. **Supplementary Table 3:** Patient demographics in Cohort 1, 2 and 3 for CTA-BET model training and testing. Pearson’s chi-squared test was used for comparing categorical variables and Kruskal Wallis test was used for comparing continuous variables between the training and test set. **Supplementary Fig. 1:** Representative cases for face detection are illustrated for rendered CTA images (from top to bottom: original CTA image, ICHSEG defacing, CTA-DEFACE defacing). The anterior point-of-view for image acquisition was maintained in each image, without considering head rotations. The probabilities of face detection by multitask cascaded convolutional neural network (MTCNN) are provided below the images, with “N/A” indicating that no face was detected. Eyes are blurred for anonymization purposes. **Supplementary Fig. 2:** Two cases demonstrating false-positive predictions by the automated vessel occlusion network (referred to as VO-ANN in this study) are displayed. In these cases, VO-ANN generated a bounding box (depicted in green) indicating a left internal carotid artery (ICA) occlusion. Both cases underwent visual examination by a radiologist with 5 years of experience. In the first case, the bounding box captured the low-contrast enhancement of the left internal jugular vein (IJV), with no occlusion observed in the left internal (ICA) or external carotid artery (ECA). In the second case, the bounding box depicted an area with calcified carotid plaque (no occlusion, no high-grade stenosis). These false-positive predictions were not observed after defacing with our CTA-DEFACE model, while reappearing after defacing with the ICHSEG model.

## Data Availability

The CTA-DEFACE model has been released as an open-source tool accompanied by thorough documentation at www.github.com/neuroAI-HD/CTA-DEFACE.

## Abbreviations

ANN: Artificial neural network
CT: Computed tomography
CTA: Computed tomography angiography
DSC: Dice similarity coefficient
GPU: Graphics processing unit
MTCNN: Multitask convolutional neural network
