# Supplementary material for: Deep learning-based defacing tool for CT angiography: CTA-DEFACE
Source: Eur Radiol Exp. 2024 Oct 9;8:111. doi: 10.1186/s41747-024-00510-9 (PMC11465008; doi:10.1186/s41747-024-00510-9)
Supplement: Supplementary file 1 — Additional file 1: Supplementary Table 1: Computed tomography angiography imaging features are depicted. Supplementary Table 2: Patient demographics in Cohort 1, 2 and 3 for CTA-DEFACE model training and testing. Pearson’s chi-squared test was used for comparing categorical variables and Kruskal Wallis test was used for comparing continuous variables between the training and test set. Supplementary Table 3: Patient demographics in Cohort 1, 2 and 3 for CTA-BET model training and testing. Pearson’s chi-squared test was used for comparing categorical variables and Kruskal Wallis test was used for comparing continuous variables between the training and test set. Supplementary Fig. 1: Representative cases for face detection are illustrated for rendered CTA images (from top to bottom: original CTA image, ICHSEG defacing, CTA-DEFACE defacing). The anterior point-of-view for image acquisition was maintained in each image, without considering head rotations. The probabilities of face detection by multitask cascaded convolutional neural network (MTCNN) are provided below the images, with “N/A” indicating that no face was detected. Eyes are blurred for anonymization purposes. Supplementary Fig. 2: Two cases demonstrating false-positive predictions by the automated vessel occlusion network (referred to as VO-ANN in this study) are displayed. In these cases, VO-ANN generated a bounding box (depicted in green) indicating a left internal carotid artery (ICA) occlusion. Both cases underwent visual examination by a radiologist with 5 years of experience. In the first case, the bounding box captured the low-contrast enhancement of the left internal jugular vein (IJV), with no occlusion observed in the left internal (ICA) or external carotid artery (ECA). In the second case, the bounding box depicted an area with calcified carotid plaque (no occlusion, no high-grade stenosis). These false-positive predictions were not observed after defacing with our CTA-DEFACE model, while reappeari [file 41747_2024_510_MOESM1_ESM.docx]

**Supplementary Appendix**

Supplementary Tables

*Supplementary Table 1:* Computed tomography angiography imaging features are depicted.

*B: body kernel; B25f, B26f, B31f, Bv40f: filtered back projection algorithm with medium smooth body kernel; I26f: iterative reconstruction algorithm with a medium smooth body kernel; H30f: medium smooth head convolution kernel. Imaging features were missing after anonymization for 10 patients in HD cohort.*

|  | **Cohort 1  (n=50)** | **Cohort 2 (n=50)** | **Cohort 3  (n=50)** |
| --- | --- | --- | --- |
| **Scanner Model** |  |  |  |
| Siemens SOMATOM Definition AS | 50% | 38% |  |
| Siemens SOMATOM X.cite |  | 18% |  |
| Siemens Sensation 16 | 30% |  |  |
| Siemens Sensation 40 |  | 44% |  |
| Philips Iqon Spectral CT |  |  | 100% |
| unknown | 20% |  |  |
| **Convolution Kernel** |  |  |  |
| B25f |  | 4% |  |
| B26f | 50% |  |  |
| B31f |  | 12% |  |
| Bv40f |  | 18% |  |
| H30f | 30% | 28% |  |
| I26f |  | 38% |  |
| B |  |  | 100% |
| unknown | 20% |  |  |
| **Slice Thickness** |  |  |  |
| 0.75 | 80% |  |  |
| 1 |  | 100% | 100% |
| unknown | 20% |  |  |
| **Exposure [mAs] median (min - max)** | 87 (73 - 105) | 62 (9 - 200) | 158 (84 - 400) |

*Supplementary Table 2:* Patient demographics in Cohort 1, 2 and 3 for CTA-DEFACE model training and testing. Pearson’s chi-squared test was used for comparing categorical variables and Kruskal Wallis test was used for comparing continuous variables between the training and test set.

*IQR: interquartile range.*

| CTA-DEFACE model | **Training Set** | | **Test Set** |  |
| --- | --- | --- | --- | --- |
|  | Cohort 1 | Cohort 2 | Cohort 3 | *p-value* |
| **Sex** |  |  |  |  |
| Male (%) | 48 | 44 | 60 | 0.23 |
| Female (%) | 52 | 56 | 40 |  |
| **Age** |  |  |  |  |
| Median (IQR) | 75 (8) | 79 (16) | 76,5 (17) | 0.63 |

*Supplementary Table 3:* Patient demographics in Cohort 1, 2 and 3 for CTA-BET model training and testing. Pearson’s chi-squared test was used for comparing categorical variables and Kruskal Wallis test was used for comparing continuous variables between the training and test set.

*IQR: interquartile range.*

| CTA-BET model | **Training Set** | | **Test Set** |  |
| --- | --- | --- | --- | --- |
|  | Cohort 1 | Cohort 2 | Cohort 3 | *p-value* |
| **Sex** |  |  |  |  |
| Male (%) | 64 | 36 | 60 | 0.42 |
| Female (%) | 36 | 64 | 40 |  |
| **Age** |  |  |  |  |
| Median (IQR) | 82 (12) | 75 (9) | 76,5 (17) | 0.07 |

Supplementary Figures


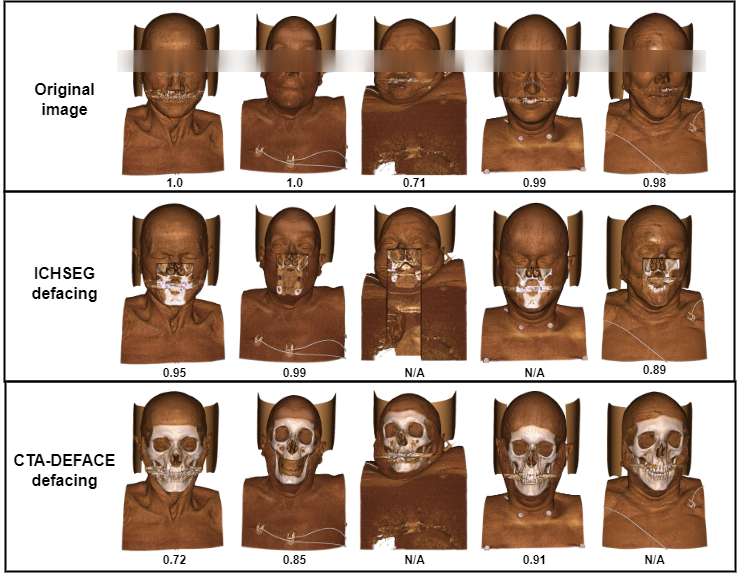
*Supplementary Figure 1:* Representative cases for face detection are illustrated for rendered CTA images (from top to bottom: original CTA image, ICHSEG defacing, CTA-DEFACE defacing). The anterior point-of-view for image acquisition was maintained in each image, without considering head rotations. The probabilities of face detection by multitask cascaded convolutional neural network (MTCNN) are provided below the images, with “N/A” indicating that no face was detected. Eyes are blurred for anonymization purposes. *CTA: computed tomography angiography.*

Supplementary Analyses

As a supplementary analysis, the impact of defacing on the CTA image was analyzed by applying the deep-learning-based vessel occlusion prediction network (Brugnara et al. 2023), referred to as VO-ANN in this study. The VO-ANN was trained to identify large vessel occlusions (LVO) in internal carotid artery (ICA), and M1-segment as well as medium vessel occlusions (MeVO) in M2- and M3-segment, which was also able to detect high-grade stenosis (HGS). Upon detecting an LVO, MeVO, or HGS, the VO-ANN generates a bounding box around the identified area for precise localization.

The effect of defacing, by both ICHSEG and CTA-DEFFACE models, on the prediction outcome of the VO-ANN algorithm was assessed. For this purpose, generated face mask areas were replaced by the 10th percentile value of the original CTA image using FSL (Jenkinson et al. 2012), effectively creating a replacement with void space. Subsequently, the VO-ANN algorithm was applied before and after defacing to compare prediction outputs.

In the case of instances processed using CTA-DEFACE, 4% revealed false-negative results, indicating that VO-ANN detected a VO in the original image but not in the defaced image. Visual examination of these cases revealed that the bounding boxes of the VO-ANN originates from false-positive predictions, implying that the detected area did not exhibit vessel occlusion. These instances are shown in the Supplementary Figure 2. Conversely, the defacing with ICHSEG did not alter the outcome prediction of the VO-ANN algorithm.


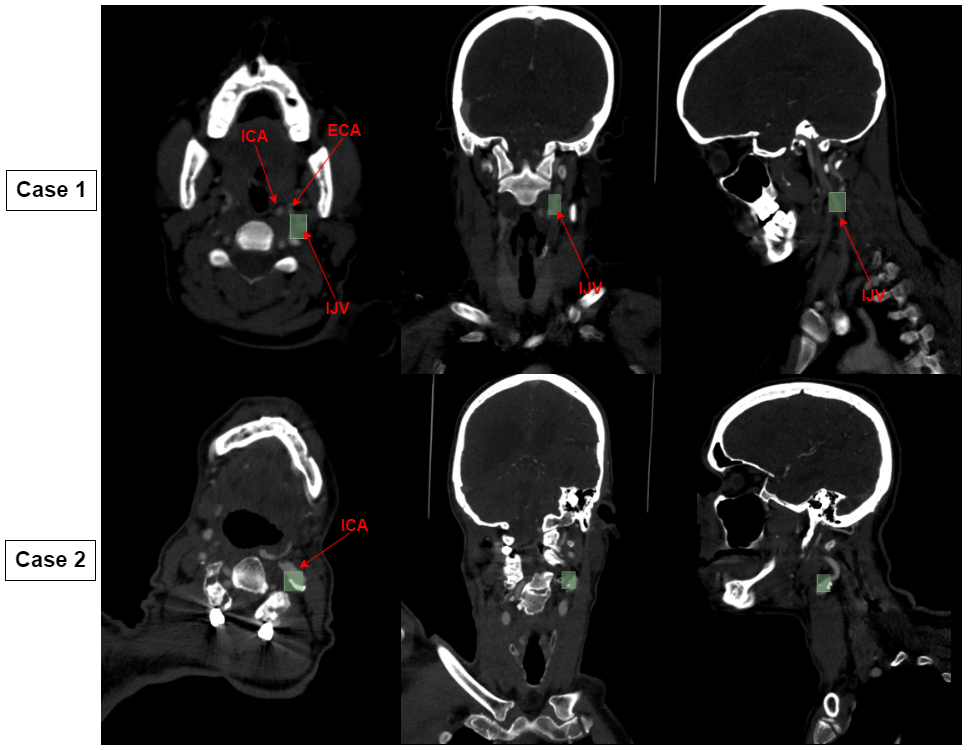


*Supplementary Figure 2: Two cases demonstrating false-positive predictions by the automated vessel occlusion network (referred to as VO-ANN in this study) are displayed. In these cases, VO-ANN generated a bounding box ( depicted in green) indicating a left internal carotid artery (ICA) occlusion. Both cases underwent visual examination by a radiologist with 5 years of experience. In the first case, the bounding box captured the low-contrast enhancement of the left internal jugular vein (IJV), with no occlusion observed in the left internal (ICA) or external carotid artery (ECA). In the second case, the bounding box depicted an area with calcified carotid plaque (no occlusion, no high-grade stenosis). These false-positive predictions were not observed after defacing with our CTA-DEFACE model, while reappearing after defacing with ICHSEG model.*

**References**

Brugnara, Gianluca; Baumgartner, Michael; Scholze, Edwin David; Deike-Hofmann, Katerina; Kades, Klaus; Scherer, Jonas et al. (2023): Deep-learning based detection of vessel occlusions on CT-angiography in patients with suspected acute ischemic stroke. In: *Nature communications* 14 (1), S. 4938. DOI: 10.1038/s41467-023-40564-8.
